# Modeling Chemotherapeutic Neurotoxicity with Human Induced Pluripotent Stem Cell-Derived Neuronal Cells

**DOI:** 10.1371/journal.pone.0118020

**Published:** 2015-02-17

**Authors:** Heather E. Wheeler, Claudia Wing, Shannon M. Delaney, Masaaki Komatsu, M. Eileen Dolan

**Affiliations:** Section of Hematology/Oncology, Department of Medicine, The University of Chicago, Chicago, IL, United States of America; University of Nebraska Medical Center, UNITED STATES

## Abstract

There are no effective agents to prevent or treat chemotherapy-induced peripheral neuropathy (CIPN), the most common non-hematologic toxicity of chemotherapy. Therefore, we sought to evaluate the utility of human neuron-like cells derived from induced pluripotent stem cells (iPSCs) as a means to study CIPN. We used high content imaging measurements of neurite outgrowth phenotypes to compare the changes that occur to iPSC-derived neuronal cells among drugs and among individuals in response to several classes of chemotherapeutics. Upon treatment of these neuronal cells with the neurotoxic drug paclitaxel, vincristine or cisplatin, we identified significant differences in five morphological phenotypes among drugs, including total outgrowth, mean/median/maximum process length, and mean outgrowth intensity (P < 0.05). The differences in damage among drugs reflect differences in their mechanisms of action and clinical CIPN manifestations. We show the potential of the model for gene perturbation studies by demonstrating decreased expression of *TUBB2A* results in significantly increased sensitivity of neurons to paclitaxel (0.23 ± 0.06 decrease in total neurite outgrowth, P = 0.011). The variance in several neurite outgrowth and apoptotic phenotypes upon treatment with one of the neurotoxic drugs is significantly greater between than within neurons derived from four different individuals (P < 0.05), demonstrating the potential of iPSC-derived neurons as a genetically diverse model for CIPN. The human neuron model will allow both for mechanistic studies of specific genes and genetic variants discovered in clinical studies and for screening of new drugs to prevent or treat CIPN.

## Introduction

The number of cancer survivors in the United States has risen to an estimated 12 million in 2012 resulting in a heightened awareness of long-term toxicities and the impact of treatment on quality of life [1]. CIPN is one of the most common and potentially permanent side effects for many anti-cancer agents and its incidence has been reported to be as high as 20–40% among all cancer patients undergoing chemotherapy [2]. General symptoms start in the fingers and toes and spread progressively up the extremities as CIPN worsens and include numbness, tingling, burning, loss of tendon reflexes and vibration sensation, and spontaneous or evoked pain [3]. There is substantial inter-patient and drug-dependent variability in time to symptom onset, time to peak symptoms, severity of peak symptoms, and reversibility [4–7]. Management is complicated by the lack of reliable means to identify at-risk patients. If patients at high risk could be identified, alternative chemotherapy regimens with similar efficacy could be considered.

In efforts to identify genetic variants associated with chemotherapeutic toxicities including CIPN, researchers have performed genome-wide association studies (GWAS) in clinical trials [8–10]. The challenges of clinical GWAS, including accurately phenotyping large patient cohorts receiving the same drug regimen and obtaining replication cohorts, have led to the development of cell based models as a complementary method to identify variants and functionally validate findings resulting from the clinical studies [11–14]. The extensively genotyped International HapMap lymphoblastoid cell line (LCL) model has been useful for this purpose and significant overlap between genetic variants associated with cellular sensitivity to paclitaxel and paclitaxel-induced clinical neuropathy has been demonstrated [15]. Follow up studies have utilized either LCLs or Neuroscreen-1 (rat pheochromocytoma) cells to functionally validate the involvement of GWAS findings in response to chemotherapeutics [15,16]. Neither cellular model represents genetically diverse human peripheral neurons, the tissue of CIPN toxicity.

In addition to clinical studies and cell line models, several rodent models have been developed to elucidate the mechanisms of CIPN and identify potential therapies, including those that measure pathological, electrophysiological, and behavioral outcomes that mimic CIPN in patients [17–20]. In particular, studies in cultured rat dorsal root ganglion (DRG) neurons have provided insight into underlying mechanisms of CIPN [21–25]. However, clinical trials that relied on preclinical animal data have not resulted in consistent benefits of candidate CIPN treatments [17,18]. Although pain reduction was observed in a recent trial of duloxetine in patients with CIPN [2], there are currently no FDA approved treatments for CIPN [3,4,26,27].

Due to the rapid advances in stem cell technology, the ability to differentiate human neurons (and other tissues) from iPSCs provides an opportunity to create panels of genetically diverse human neurons. Large quantities of neurons from one iPSC line (iCell Neurons) are commercially available for preliminary assay development, drug screens, siRNA screens or functional studies of candidate genes. Upon treatment of iCell Neurons with increasing concentrations of representative neurotoxic agents (paclitaxel, vincristine or cisplatin), we identified reproducible decreases in neurite outgrowth phenotypes. As a proof of concept, we show that decreased expression of the paclitaxel target *TUBB2A* by siRNA transfection causes decreased neurite outgrowth after paclitaxel treatment, as expected based on a previous patient study [28]. We show that the variance in neurite outgrowth phenotypes is greater between individuals than the experimental variance within individuals, demonstrating that larger genetic association studies are possible with iPSC-derived neurons.

## Materials and Methods

### iCell neuron culture

Neurons derived from human induced pluripotent stem cells (iCell Neurons and MyCell Neurons reprogrammed from LCLs) were purchased from Cellular Dynamics International (Madison, WI, USA). Neurons were maintained according to the manufacturers protocol. Depending on the experiment, 1.33 x 10^4^ cells/well or 4 x 10^4^ cells/well were seeded using either a single coating or double coating plating method. Drug treatment only experiments used the single coating method where 96-well black, clear bottom Greiner Bio-One plates (Monroe, NC, USA) were pre-treated with 0.01% poly-L-ornithine and coated with 3.3 μg/ml laminin (Sigma-Aldrich; St.Louis, MO, USA) prior to seeding. siRNA experiments used the double coating method where cells were mixed with 3.3 μg/ml laminin prior to seeding on poly-D-lysine coated 96-well Greiner Bio-One plates at a density of 1.33 x 10^4^ cells/well. For experiments presented in the Results, cells were either treated with drug or transfected with siRNA 4 h after plating. Some experiments presented in the Supporting Information evaluated treatment of drugs at 1, 2, 3, 5, or 11 d following plating.

### Drug preparation

Paclitaxel (Sigma-Aldrich) was prepared in the dark by dissolving powder in 100% DMSO and filtered to obtain a stock solution of 58.4 mM. Stock drug was serially diluted in media for final dosing concentrations ranging from 0.001 μM to 100 μM, increasing by factors of ten. Control wells were treated with 0.17% final concentration of DMSO to match drug treatments. Vincristine (Development Therapeutics Program at NCI or Sigma-Aldrich) was prepared on ice in the dark by dissolving powder in cold PBS and filtered to obtain a stock solution of 100 mM. Stock drug was individually diluted in media and added to the cells in a range of 0.001 μM to 100 μM. Cisplatin (Sigma-Aldrich) was prepared in the dark by dissolving powder in 100% DMSO and filtered to obtain a stock solution of 20 mM. Stock drug was serially diluted in media for final dosing concentrations ranging from 0.001 μM to 100 μM. Control wells were treated with 0.2% or 0.5% final concentration of DMSO to match drug treatment. Hydroxyurea (Sigma-Aldrich) was prepared by dissolving powder in PBS and filtered to obtain a stock solution of 1 M. Stock drug was serially diluted in media for final dosing concentrations ranging from 0.001 μM to 100 μM.

### Cell viability and apoptosis assays

Cell viability 72 h after drug treatment was assessed using the CellTiter-Glo assay (Promega, Madison, WI, USA), which measures ATP levels. Apoptosis induction 48 h after drug treatment was assessed using the Caspase-Glo 3/7 assay (Promega). Four replicates of the viability assay and five replicates of the apoptosis assay were performed. At least two wells per drug dose were measured in each replicate.

### High content imaging and neurite outgrowth analysis

After drug treatments of 48 or 72 h, neurons were stained for 15 minutes at 37°C with 1 μg/ml Hoechst 33342 (Sigma-Aldrich), 2 μg/ml Calcein AM and 1 μM Ethidium Homodimer-2 (Molecular Probes, Life Technologies Inc., Carlsbad, CA, USA) then washed twice using dPBS without calcium or magnesium (LifeTechnologies). Imaging was performed at 10x magnification using an ImageXpress Micro (Molecular Devices, LLC, Sunnyvale, CA, USA) at the University of Chicago Cellular Screening Center. Individual cell measurements of mean/median/maximum process length, total neurite outgrowth (sum of the length of all processes), number of processes, number of branches, cell body area, mean outgrowth intensity, and straightness were calculated using the MetaXpress software Neurite Outgrowth Application Module (Molecular Devices, LLC). At least 500 cells per dose (except 100 μM) were quantified and three replicates of each drug treatment were performed. The mean number of cells quantified per well (3–4 wells/dose) is shown for each experiment in S1 Table. Cell level outgrowth data is available for every experiment presented in the Results in S2–S6 Tables. For each replicate, the mean value relative to control at each dose was used to calculate the area under the concentration curve (AUC) for each of the nine phenotypes. For analyses comparing the four drugs, AUCs were calculated from 0.001–10 μM or from 0.001–100 μM and for analyses comparing the four LCL-iPSC-derived neuronal cells, AUCs were calculated from 0.001–100 μM. The differences in AUCs among the four drugs or among the four individuals for each phenotype were tested for significance by one-way ANOVA using the *oneway.test* function in R version 3.0.2, not assuming equal variances. For each replicate, we fit a linear model (*outgrowth* = log_10_(*dose*)) to determine the effect sizes of the dose response for relative total outgrowth using the *lm* function in R.

### Time-lapse video collection

iCell Neurons were plated at a density of 1.33 x 10^5^ cells/ml into MatTex 35mm glass-bottom dishes (MatTek, Ashland, MA, USA) with 14 mm insert coated with 3.3 μg/ml laminin. Imaging was performed at the University of Chicago Integrated Light Microscopy Facility. Images were captured with an Olympus VivaView incubator-based, epifluorescence microscope (Olympus Corporation of the Americas, Center Valley, PA, USA) run by MetaMorph software (Molecular Devices, LLC) using the 40x objective at 10 min intervals for 28 h. The cells were allowed to grow for 4 h before 10 μM of each drug or 0.17% DMSO vehicle control was added. Imaging began 1 h prior to drug treatment. The image stacks were prepared into videos using ImageJ software [29].

### siRNA

iCell Neurons underwent siRNA transfection with Dharmacon Accell technology (Thermo Fisher Scientific Inc., Waltham, MA, USA). Accell human *TUBB2A* SMARTpool siRNA was diluted to a stock concentration of 100 μM in 1X siRNA Buffer (ThermoScientific) and then diluted to 1 μM in Accell siRNA delivery media (ThermoScientific). A non-targeting siRNA pool from ThermoScientific was used as a negative control. Four hours after plating, the iCell neuron maintenance media was removed and the Accell delivery media was added for 24 h. At the 24 h timepoint, cells were then treated with 0.1 μM paclitaxel or DMSO vehicle control as described above. High content imaging was performed 24 h after paclitaxel treatment. The entire experiment was replicated three times. Quantitative reverse transcription polymerase chain reaction (qRT-PCR) was performed to measure the level of expression of *TUBB2A* (Hs00742533_s1) 24 and 48 h after transfection. Two wells of 1.33 x 10^4^ cells/well were lysed and prepared for qRT-PCR using the Cells to CT kit from Life Technologies. A comparative delta delta CT method was used with human beta-2-microglobulin (NM_004048.2) as the endogenous control to determine the percent of knockdown at each given timepoint compared to non-targeting control (NTC). Each sample was reverse transcribed twice and run in triplicate on the Life Technology Viia7 PCR machine. The differences in mean relative total outgrowth (0.1 μM paclitaxel:vehicle) between *TUBB2A* siRNA and NTC were tested for significance by Welch's t-test.

### LCL reprogramming and neuronal differentiation

The two most paclitaxel-sensitive (GM12814, GM12892) and two most paclitaxel-resistant (GM07022, GM12752) unrelated LCLs from the CEU HapMap population (HAPMAPPT01, Northern and Western European ancestry from Utah) were chosen for reprogramming into iPSC cells. The lines were chosen by ranking all CEU LCLs with paclitaxel-induced cytotoxicity (viability) and caspase-3/7 measurements [30] by sensitivity and choosing the two with the highest mean rank and the two with the lowest mean rank (S7 Table). These four LCLs were purchased and sent directly from the Coriell Cell Repository (Camden, New Jersey, USA) to Cellular Dynamics International (CDI) for reprogramming and differentiation into neurons via their MyCell Neurons product. CDI generated EBV-free iPSCs from the LCLs using their feeder-free episomal method [31] and subsequently differentiated the iPSCs into neuronal cells for use in the chemotherapy treatment experiments. The neuronal cells are differentiated along a developmental pathway to produce cortical neurons (verified by *DACH1, FOXG1* and *OTX2* gene expression). Their purity was assessed by an intracellular flow cytometry assay for Tuj1 (βIII-tubulin) positive and nestin negative cells (>97% for all lines, see S7 Fig.). The neuronal cells from CDI do not contain glia or any other proliferating cell type. The differentiated neurons were named N12814, N12892, N07022 and N12752, according to their LCL identification number. We verified the identity of the neurons, the LCLs from Coriell used to generate iPSCs, and the four LCLs stored in our laboratory by genotyping the 47 informative SNPs included in the Sequenom iPLEX Sample ID Plus Panel (Sequenom, Inc., San Diego, CA). DNA was extracted from each sample using the DNeasy Blood and Tissue Kit (Qiagen, Valencia, CA). Genotyping was performed following the iPLEX Pro application guide and the iPLEX Pro reaction products were dispensed onto a 384-sample SpectroCHIP and run on a Sequenom MassARRAY system at The University of Chicago Comprehensive Cancer Center DNA Sequencing and Genotyping Facility. Genotypes passed quality control (formed clear genotype clusters) for 46/47 SNPs and all 46 genotypes matched the known HapMap (http://hapmap.ncbi.nlm.nih.gov/) genotypes for all samples.

## Results

### Differences in neurite outgrowth across chemotherapeutics

**Fig 1:**
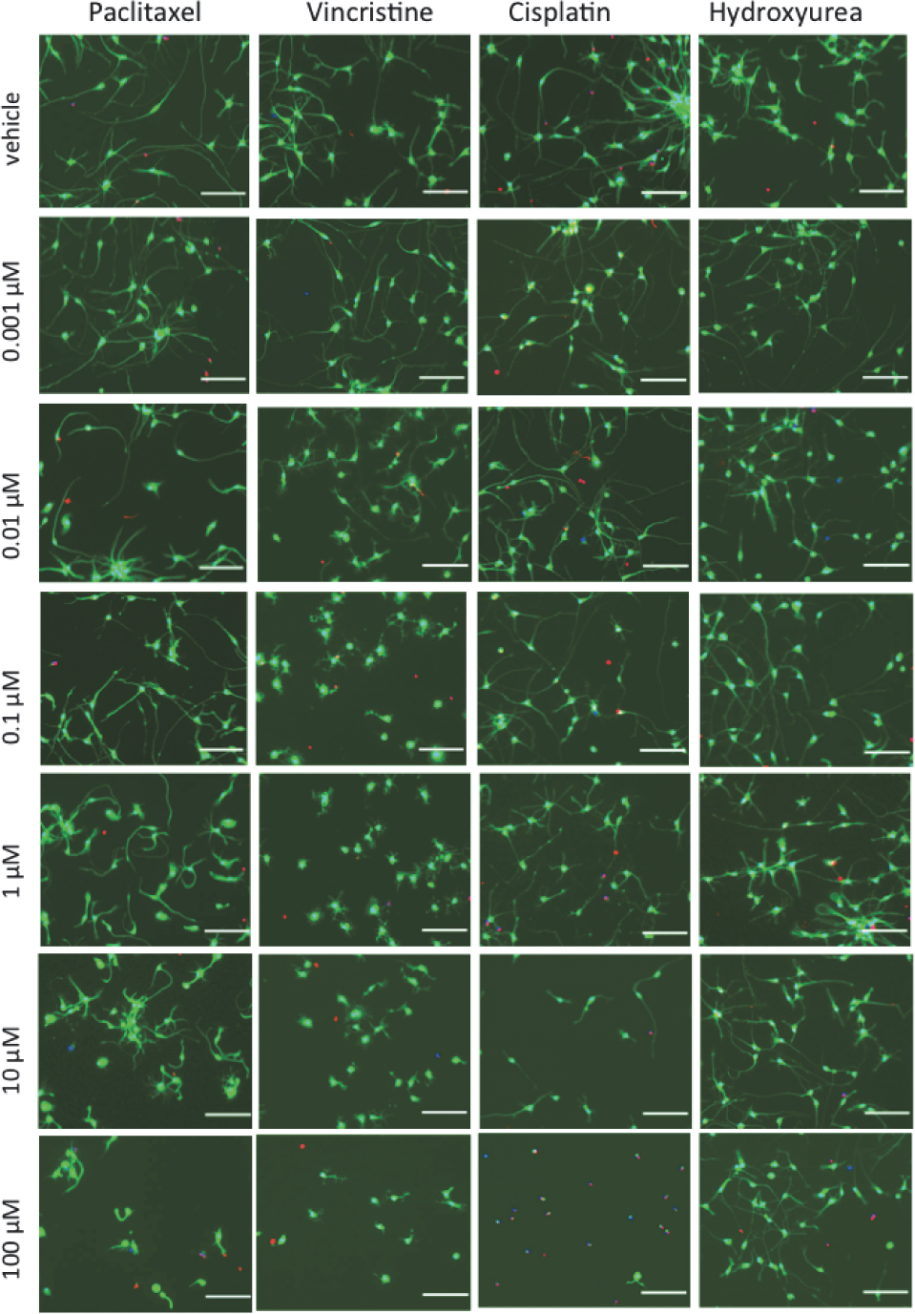
Representative images from iCell Neuron chemotherapeutic treatment. Representative micrographs comparing neurite outgrowth of iCell Neurons upon 72 h treatment with the neuropathy-inducing drugs paclitaxel, vincristine, and cisplatin and the non-neuropathy-inducing negative control hydroxyurea. Images taken at 10x magnification, scale bar = 100 μm, green = Calcein AM (cell-permeant stain, live cells), blue = Hoechst 33342 (cell-permeant, nucleic acid stain, live cell nuclei), red = Ethidium Homodimer-2 (cell-impermeant, nucleic acid stain, dead cells).

**Fig 2:**
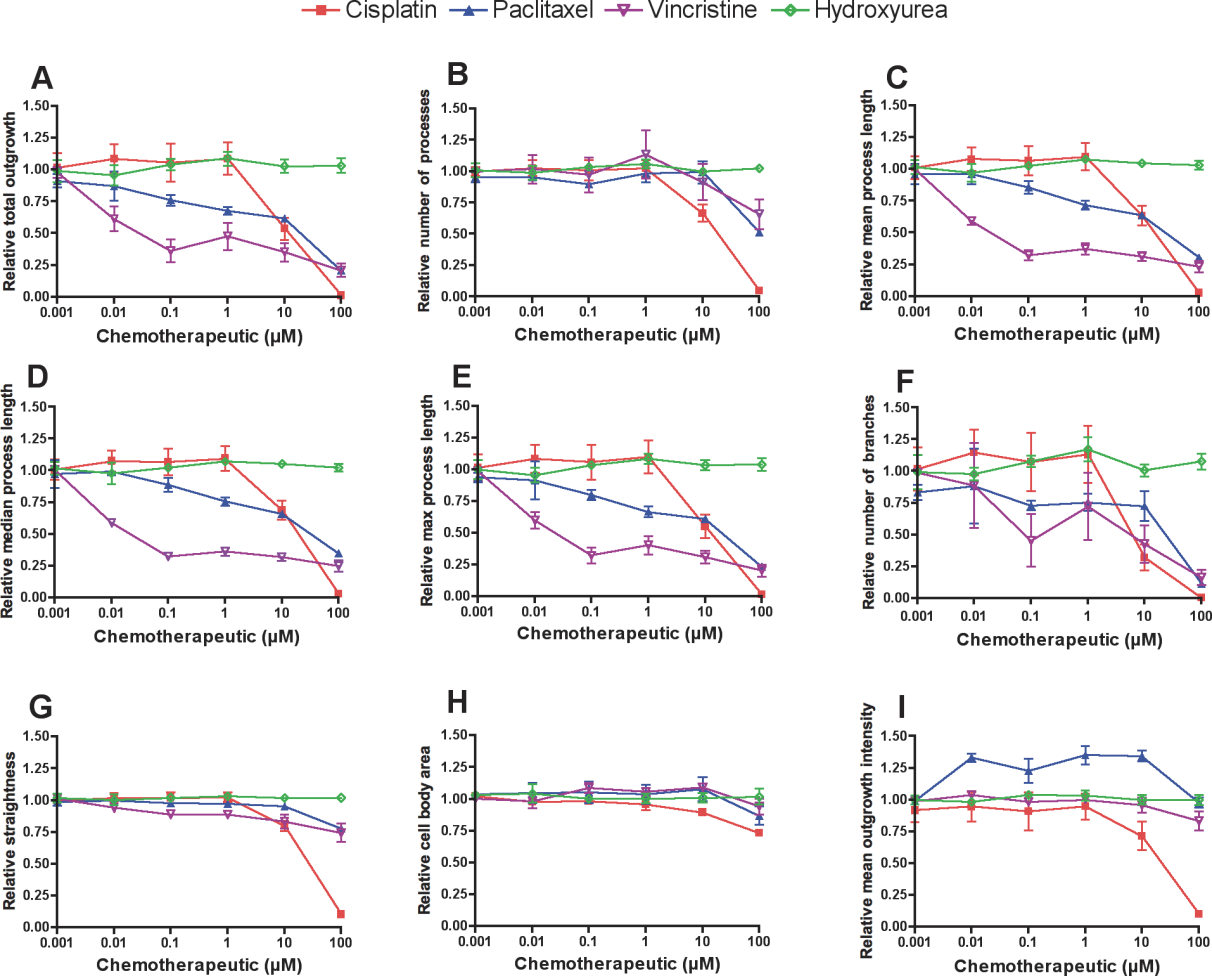
Neurite outgrowth response upon chemotherapeutic treatment of iCell Neurons. Nine phenotypes measured after 72 h of drug exposure were tested for differences across drugs by one-way ANOVA of the area under the curve (AUC) calculated from 0.001–10 μM including relative (A) total outgrowth (P = 0.014), (B) number of processes (P = 0.623), (C) mean process length (P = 0.005), (D) median process length (P = 0.002), (E) maximum process length (P = 0.009), (F) number of branches (P = 0.065), (G) straightness (P = 0.081), (H) cell body area (P = 0.176), and (I) mean outgrowth intensity (P = 0.005). Error bars represent the standard error of the mean from three independent experiments of >500 cells per dose.

Several preliminary studies were done to optimize plating density, time of outgrowth prior to drug treatment, drug treatment length and drug concentrations in order to achieve reproducible dose-response curves for neurite outgrowth phenotypes for 4 distinct chemotherapeutic drugs: vincristine, paclitaxel, cisplatin and hydroxyurea (S1–S3 Figs.). To assess morphological changes of 4 different chemotherapeutics, we treated human neurons derived from iPSCs (iCell Neurons) with increasing concentrations of drug for 72 h and measured neurite outgrowth phenotypes by high content imaging. For the three drugs that cause peripheral neuropathy (paclitaxel, vincristine, cisplatin), we observed variable decreases in the neurite process phenotypes upon increasing drug concentration (Fig. 1). As illustrated in Fig. 2A, across three replicate experiments, vincristine caused the largest decrease in relative total outgrowth (β = -0.14 ± 0.021), followed by (paclitaxel β = -0.11 ± 0.0053), then cisplatin (β = -0.069 ± 0.013). When iCell Neurons were treated with a fourth chemotherapeutic drug, hydroxyurea, a negative control not known to cause neuropathy, there was no decrease in relative total outgrowth (β = 0.0042 ± 0.0023). Among the neurotoxic drugs, in addition to these differences in linear regression effect sizes, we observed that the cisplatin dose-response was the least linear. Neurite outgrowth phenotypes remained constant until the 1 μM cisplatin dose and then dropped sharply (Fig. 2).

Relative mean outgrowth intensity increased upon iCell Neuron treatment with paclitaxel doses from 0.01–10 μM (Fig. 2I), which is represented by thicker, brighter neurites in paclitaxel-treated cells compared to those treated with the other drugs (also see Fig. 1). This thickening of neurites was visible in time-lapse photography of the iCell Neurons upon treatment with 10 μM paclitaxel (S1 Movie). While the addition of cisplatin or paclitaxel appeared to slow the outgrowth of iCell Neurons over 28 h compared to vehicle or hydroxyurea (negative control), a visible retraction of neurite processes was observed upon treatment with 10 μM vincristine (S1 Movie).

**Fig 3:**
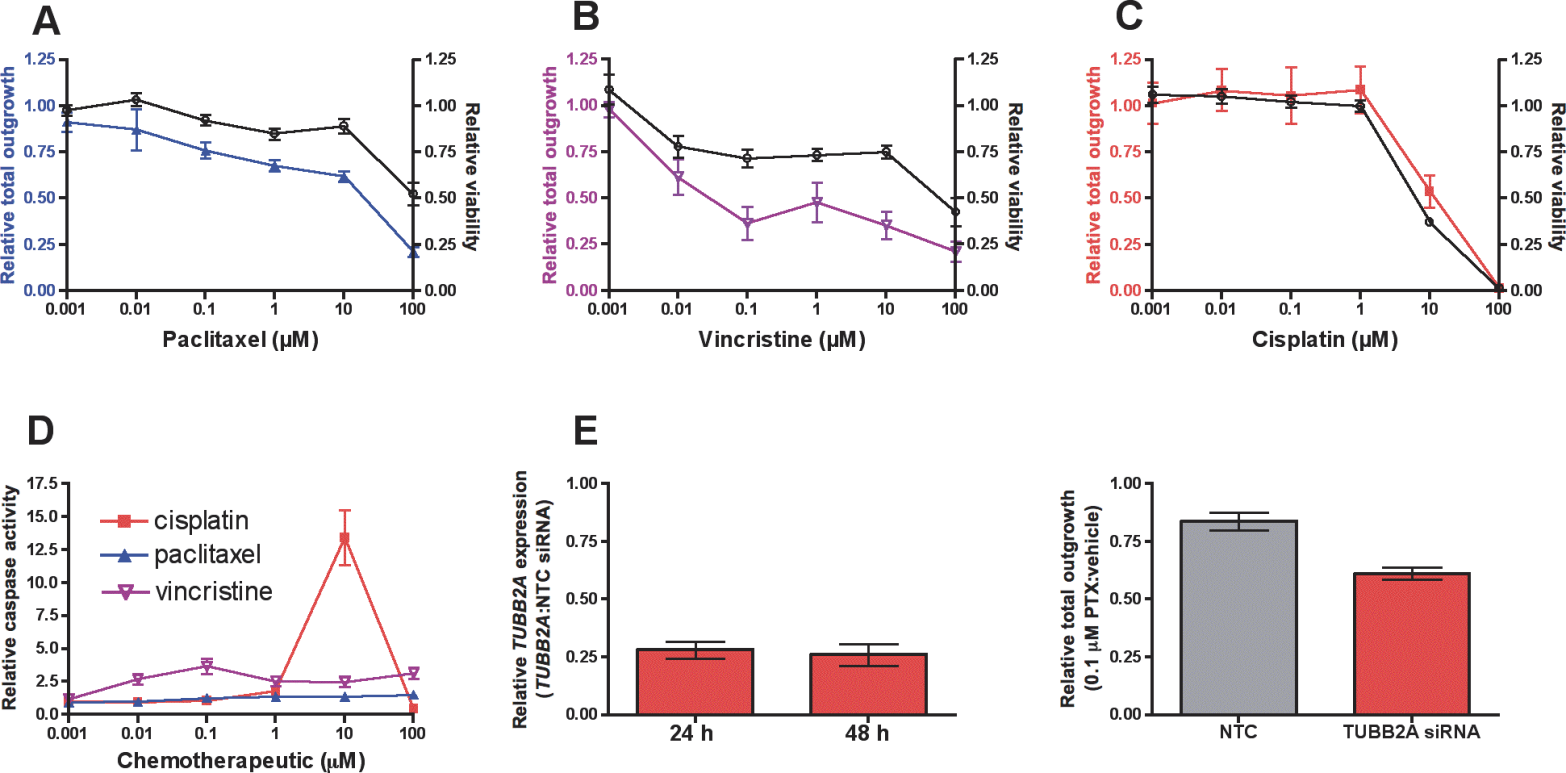
Neurite outgrowth, viability, and apoptotic response upon chemotherapeutic treatment of and gene perturbation of *TUBB2A* in iCell Neurons. Relative viability measured by CellTiter-Glo (black lines, right Y-axes) was significantly higher than relative total outgrowth (colored lines, left Y-axes) upon treatment of iCell Neurons for 72 h with (A) paclitaxel (Welch's t-test of AUC P = 0.0005) and (B) vincristine (P = 0.027), but not (C) cisplatin (P = 0.71). Three replicates of the total outgrowth assay and four replicates of the viability assay were performed. (D) Relative caspase 3/7 activity measured by Caspase-Glo 3/7 after 48 h treatment significantly differed among drugs (one-way ANOVA of AUC calculated from 0.001–10 μM P = 0.002). Five replicates of the apoptosis assay were performed. (E) Paclitaxel-induced decrease in neurite outgrowth of iCell Neurons is influenced by *TUBB2A* expression. Left, relative *TUBB2A* expression 24 to 48 h post-transfection. Right, decreased expression of *TUBB2A* by siRNA transfection causes a greater decrease in relative total neurite outgrowth of iCell Neurons (Welch's t-test P = 0.011) 24 h post-treatment with 0.1 μM paclitaxel (48 h post-transfection). Error bars represent the standard error of the mean from three independent transfection experiments. NTC = non-targeting control.

For the nine phenotypes measured by the MetaXpress high content imaging software, we calculated the area under the concentration curve (AUC) for each of the three replicates and used these values to test for differences among the four drugs by ANOVA. When all data points are included (up to100 μM) in the AUC calculation, all nine phenotypes showed a significant difference among the four drugs and seven phenotypes significantly differed among the three neurotoxic drugs (P < 0.05, S8 Table). A more conservative approach excludes the 100 μM dose in the AUC calculation because of the greatly reduced viability among the neurotoxic drugs, cisplatin in particular, at this dose (Fig. 3A-C). In this case, five of the nine phenotypes, including relative total outgrowth, mean/median/maximum process length, and mean outgrowth intensity, showed a significant difference in mean AUC among drugs (P < 0.05), while relative number of processes, number of branches, straightness, and cell body area did not differ among drugs (Table 1, Fig. 2).

**Table 1:** ANOVA results comparing the AUCs of relative neurite outgrowth phenotypes among 4 drugs after 72 h treatment of iCell Neurons considering 0.001 to 10 μM.

Phenotype	Paclitaxel AUC (SE)	Vincristine AUC (SE)	Cisplatin AUC (SE)	Hydroxyurea AUC (SE)	F stat	P value
total outgrowth	6.24 (0.02)	5.64 (0.20)	6.51 (0.18)	6.68 (0.06)	16.64	0.014
number of processes	6.57 (0.06)	6.63 (0.13)	6.53 (0.10)	6.67 (0.04)	0.65	0.623
mean process length	6.35 (0.004)	5.54 (0.09)	6.57 (0.14)	6.68 (0.04)	37.24	0.005
median process length	6.40 (0.02)	5.54 (0.07)	6.59 (0.13)	6.69 (0.03)	51.48	0.002
maximum process length	6.27 (0.02)	5.54 (0.15)	6.52 (0.18)	6.67 (0.05)	20.46	0.009
number of branches	6.27 (0.06)	5.85 (0.40)	6.33 (0.33)	6.70 (0.08)	5.61	0.065
straightness	6.61 (0.02)	6.51 (0.03)	6.60 (0.05)	6.67 (0.03)	4.6	0.081
cell body area	6.71 (0.08)	6.70 (0.02)	6.59 (0.03)	6.67 (0.04)	2.69	0.176
mean outgrowth intensity	6.95 (0.02)	6.63 (0.03)	6.44 (0.20)	6.66 (0.04)	23.16	0.005

ANOVA = one-way analysis of variance (not assuming equal variances), SE = standard error, AUC = area under the concentration curve calculated from 0.001–10 μM.

For paclitaxel and vincristine, the observed decrease in total neurite outgrowth over 72 h of treatment did not coincide with a dramatic decrease in cell viability as measured by ATP levels (Fig. 3A-B). However, overlapping curves depicting the decrease in total outgrowth and the decrease viability upon cisplatin treatment were observed, indicating cell death is the likely cause of the cisplatin-induced neurite outgrowth reduction (Fig. 3C). Apoptosis following DNA damage as demonstrated by the 13-fold increase in caspase-3/7 activity 48 h after treatment with 10 μM cisplatin dose plays an important role in cisplatin-induced cell death (Fig. 3D). A smaller, approximately 3-fold increase in caspase-3/7 activity was observed at doses of vincristine above 0.01 μM 48 h after treatment, corresponding to a slight decrease in viability at 72 h (Fig. 3B,D). Interestingly, paclitaxel did not cause caspase-3/7 activation (Fig. 3D).

### Reduced TUBB2A expression sensitizes neurons to paclitaxel

We tested several variables including transfection reagents, plating density and plate coating in an attempt to optimize conditions for siRNA transfection in iCell Neurons (S4–S5 Figs.). Paclitaxel binds to β-tubulin to exert its cytotoxic effect and genetic variants within the promoter of *TUBB2A* have previously been shown to be associated with increased expression of the gene and reduced risk of paclitaxel-induced peripheral neuropathy [28]. Using optimized transient siRNA transfection conditions, we decreased expression of *TUBB2A* resulting in increased sensitivity of iCell Neurons to 0.1 μM paclitaxel, as measured by reduced total neurite outgrowth (P = 0.011, Fig. 3E). This decreased expression of *TUBB2A* resulted in a 0.23 ± 0.06 decrease in relative total neurite outgrowth.

### Differences in neurite outgrowth across genetically distinct cell lines

**Fig 4:**
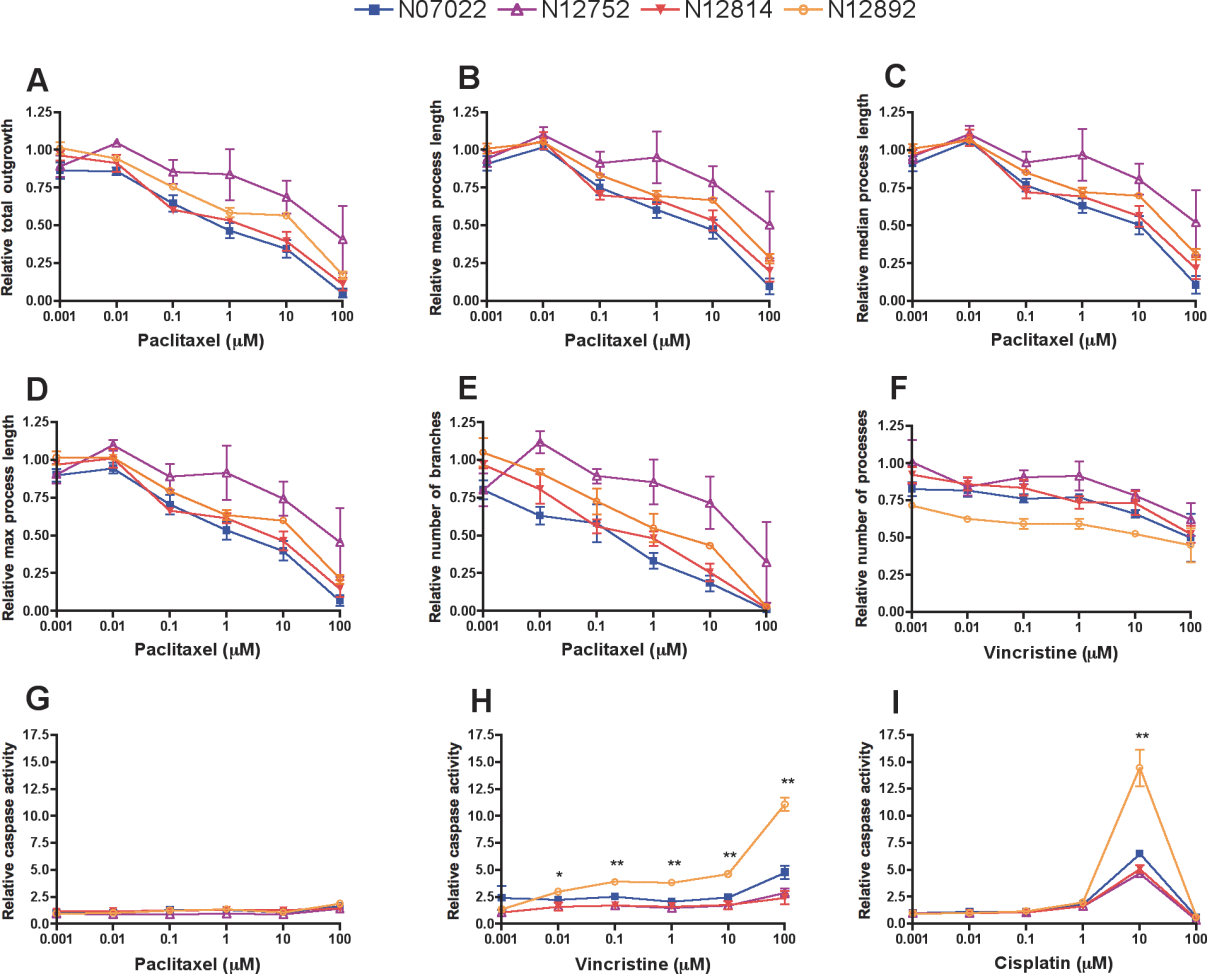
Neurite outgrowth and apoptotic response upon chemotherapeutic treatment of 4 genetically distinct LCL-iPSC-derived Neuron lines. Phenotypes measured after 72 h of drug exposure that significantly differed across individuals (ANOVA of AUC calculated from 0.001–100 μM) include relative paclitaxel-induced (A) total outgrowth (P = 0.005), (B) mean process length (P = 0.019), (C) median process length (P = 0.022), (D) maximum process length (P = 0.009), (E) number of branches (P = 0.018), and vincristine-induced (F) number of processes (P = 0.022). Relative caspase 3/7 activity measured by Caspase-Glo 3/7 after 48 h treatment of (G) paclitaxel, (H) vincristine, and (I) cisplatin. Doses of drug that caused a mean greater than 2-fold increase in caspase activity relative to control were tested for significant differences among individual cell lines by one-way ANOVA, *P < 0.05 and **P < 0.001. Error bars represent the standard error of the mean from three independent experiments of >500 cells per dose.

To determine whether phenotypes for a given drug differed among genetically diverse neurons, iPSCs reprogrammed from four LCLs were differentiated into neurons and neurite outgrowth phenotypes were measured via high content imaging for each neuronal line upon treatment for 72 h with paclitaxel, vincristine, or cisplatin. The two most sensitive and two most resistant unrelated LCLs from the CEU HapMap population based on paclitaxel-induced cytotoxicity and caspase-3/7 data [30] were chosen for reprogramming into iPSCs. For the nine phenotypes measured by the MetaXpress high content imaging software, we calculated the area under the concentration curve (AUC) from 0.001–100 μM for each of the three replicates and used these values to test for greater variance among than within individuals per drug by ANOVA. For paclitaxel-treated neurons, five of the nine phenotypes, including relative total outgrowth, mean/median/maximum process length, and number of branches, significantly differed among individuals (ANOVA P < 0.05, Table 2, Fig. 4A-E). For vincristine-treated neurons, the relative number of processes significantly differed among individuals (ANOVA P < 0.05, Table 2, Fig. 4F). None of the nine outgrowth phenotypes significantly differed among cisplatin-treated neurons (Table 2). Negligible (less than 2-fold change) caspase-3/7 activity was detected in the paclitaxel-treated neurons (Fig. 4G), consistent with the apoptosis results in the iCell Neurons (Fig. 3D). Mean caspase-3/7 activity across the neurons was greater than 2-fold higher than the control upon treatment with 0.01–100 μM vincristine and significantly differed among individuals at these doses (ANOVA P < 0.05, Fig. 4H). Similar to that observed with iCell Neurons, cisplatin-induced caspase-3/7 activity was highest at the 10 μM dose in the LCL-derived neurons, ranging from 4.5-fold to 14.5-fold increases relative to control, which represents a significant difference among individuals (ANOVA P < 0.001, Fig. 4I).

**Table 2:** ANOVA results comparing the AUCs of relative neurite outgrowth phenotypes among 4 genetically different LCL-iPSC-derived Neuron lines after 72 h drug treatment.

	Paclitaxel		Vincristine		Cisplatin	
Phenotype	F stat	P value	F stat	P value	F stat	P value
total outgrowth	23.07	0.005	5.49	0.080	4.98	0.085
number of processes	4.01	0.112	9.60	0.022	1.29	0.393
mean process length	11.64	0.019	3.20	0.161	6.27	0.059
median process length	10.43	0.022	3.45	0.146	5.30	0.082
maximum process length	17.79	0.009	3.68	0.139	6.04	0.054
number of branches	11.03	0.018	4.06	0.113	2.29	0.218
straightness	1.66	0.312	1.78	0.280	0.40	0.763
cell body area	3.62	0.115	4.46	0.094	2.46	0.21
mean outgrowth intensity	3.04	0.151	2.53	0.200	0.34	0.796

ANOVA = one-way analysis of variance (not assuming equal variances), AUC = area under the concentration curve calculated from 0.001–100 μM.

## Discussion

We have applied a human neuronal cell model to the study of chemotherapeutic neurotoxicity. We demonstrate reproducible differences in morphological changes including neurite outgrowth phenotypes, cellular viability and apoptosis among four distinct chemotherapeutic drugs. Importantly, we identified differences among genetically distinct iPSC-derived neurons in the degree of apoptosis for vincristine and cisplatin, relative number of processes for vincristine and relative total outgrowth, process length, and number of branches for paclitaxel. The iPSC-derived neurons are a highly relevant human model currently available for neurotoxicity and much improved from the LCL model used previously for screening and validation.

In the human neuronal model, vincristine was the most neurotoxic as measured by morphological changes following treatment. Similarly, in patients, neurotoxic doses of vincristine are approximately 40-fold lower than those for paclitaxel and 75-fold lower than those for cisplatin [32]. Cisplatin-induced neuropathy is also known to have delayed onset, often not appearing until several months after treatment has been completed and thought to be due to an accumulation of drug [32–34]. Importantly we see phenotypic changes in our human neuronal model at physiologically relevant plasma concentrations seen in patients: 10–100 nM for paclitaxel [35], 1–100 nM for vincristine [36], and 1–10 μM for cisplatin [37].

The human neurons used in this study are not peripheral neurons, but are predominantly glutamatergic and GABAergic cortical neuronal subtypes. Researchers have created iPSC-derived neurons to study neuronal diseases and have found these cells to be representative to the neuronal disease [38–40]. The expectation is that large quantities of peripheral neurons will undergo production at some point. Despite this shortcoming, having a highly pure, readily available human neuron is a significant advance relative to the tools of the past. Furthermore, the human neuronal model complements the rat DRG model and offers the advantage that the human model will better reflect the complex genetic interactions that result in neurotoxicity in humans.

Prior animal studies reveal similar differences among drugs compared to our results. For instance, in a study of rat DRG neurons treated with the same three neurotoxic drugs, vincristine had the lowest IC_50_ for neurite outgrowth, the paclitaxel IC_50_ was intermediate, and the cisplatin IC_50_ was the highest [23]. Consistent with vincristine being the most severe, vincristine treatment decreased both anterograde and retrograde fast axonal transport in isolated squid axoplasm, whereas paclitaxel only decreased anterograde transport [41]. Also, only higher doses of cisplatin reduced caudal nerve conduction velocity in BALB/c mice, consistent with our finding of no outgrowth reduction in human neurons until high doses of cisplatin [42].

Our findings support different mechanisms of action among the three neurotoxic drugs examined. The known cisplatin mechanism of action of DNA platination with eventual apoptosis is consistent with our findings in iPSC-derived neurons of morphological changes concomitant with caspase 3/7 activation. We saw the largest caspase 3/7 activity at the 10 μM cisplatin dose, but did not detect any caspase 3/7 activity at the 100 μM dose, likely because the activation had already occurred prior to the 48 h time point, leaving few living cells for caspase measurement (S1 Table). Consistent with our observation that cisplatin-treated iPSC-derived neurons have the largest apoptotic response among drugs tested, post-mitotic rat dorsal root ganglion neurons attempted to re-enter the cell cycle and underwent apoptosis upon cisplatin treatment [21]. Paclitaxel may be disrupting mitochondrial function more than the other two drugs [43,44], suggesting mitochondrial function in iPSC-derived neurons might be a worthwhile phenotype to investigate. Because we observed large, consistent dose-response effects with little cell death, the neurite total outgrowth and process length phenotypes are most appropriate for future studies of paclitaxel- and vincristine-induced peripheral neuropathy in the human iPSC-derived neuron model. Cisplatin, on the other hand, may be best studied in the neuronal model using apoptotic or additional, yet untested, phenotypes.

The induced pluripotent stem cell derived human neuronal model is one of the most appropriate cell types available for follow-up functional studies from patient CIPN GWAS. As a proof of concept, we show that decreased expression of the gene encoding paclitaxel target TUBB2A causes decreased neurite outgrowth after paclitaxel treatment. This result is concordant with findings from a previous patient study connecting decreased expression of the gene with promoter alleles that associate with increased risk of paclitaxel-induced peripheral neuropathy [28]. In a recent GWAS of vincristine-induced peripheral neuropathy in pediatric acute lymphoblastic leukemia patients, a promoter SNP of *CEP72* was genome-wide significantly associated with neuropathy risk [45]. In that study, we used the human iPSC-derived neuron model to show that decreased expression of *CEP72* decreased the relative total outgrowth, number of processes, and number of branches upon vincristine treatment, which greatly supported additional functional findings [45]. Importantly, this model will have utility for functional validation of other genes associated with CIPN found in GWAS or other genomic studies.

While GWAS for CIPN have revealed a few promising associations [8,10,45], the problem has been in identifying large replication patient cohorts receiving the same drug regimen for replication. Indeed, replication studies in oncology are extremely challenging because a large, well-controlled trial using the same drug regimen is rarely performed twice [46]. Here, we show that the variance in neurite outgrowth phenotypes is greater between than within individuals, demonstrating the potential of larger genetic association studies using the human iPSC-derived neuron model. A recent study demonstrated that genetic background was the major cause of transcriptional variation among iPSC lines, suggesting that future studies should focus on collection of a large number of donors, rather than generating large numbers of lines from the same donor [47].

While the two most paclitaxel-sensitive and two most paclitaxel-resistant LCLs [30] were chosen for reprogramming into iPSCs, the differences among neurite outgrowth phenotypes in derived neurons, while statistically significant, were not so dramatic. Indeed, one of the resistant lines in LCLs (GM07022) was one of the most paclitaxel-sensitive lines in neurons (N07022), while GM12752/N12752 was paclitaxel-resistant in both cell types (Fig. 4A, S6 Fig.). These differences suggest there may be neuron-specific mechanisms of drug sensitivity not present in the LCL model, which should be studied in larger populations of neurons. As there is a serious need for a more relevant and genetically diverse human cellular model for studies of drug toxicity, the use of differentiated cells (cardiomyocytes, hepatocytes, neurons) will have great implications for the field of pharmacogenomics. These studies provide a framework for the discovery, validation, and identification of 1) individuals at high genetic risk for neurotoxicity; 2) genetic components and genes contributing to CIPN and; 3) druggable targets to treat or prevent this devastating side effect of chemotherapy.

## Supporting Information

S1 Fig Determination of cell density for high content imaging and neurite outgrowth analysis of iPSC-derived neurons. Representative images of iCell Neurons plated at densities of (a) 4 x 10^4^ cells/well and (b) 1.33 x 10^4^ cells/well stained with Calcein AM (green) and analyzed by the MetaXpress software Neurite Outgrowth Application Module (red). Note that at the higher density, clusters of neurons produced regions not accessible for MetaXpress to call cells and measure neurite outgrowths (cells were either missed or given a value of zero for process length measurements).
(DOCX)

S2 Fig Determination of iCell Neuron outgrowth time prior to drug treatment for high content imaging and neurite outgrowth analysis. Allowing 4 h of neurite outgrowth prior to 72 h paclitaxel treatment resulted in consistent dose-response curves for relative total outgrowth (n = 3). Allowing 3–11 d of neurite outgrowth prior to either 48 or 72 h PTX treatment did not decrease total outgrowth upon increasing doses (n = 1). Allowing 1 d of neurite outgrowth prior to 48 h PTX treatment may result in consistent dose-response curves for relative total outgrowth, but was only tested once (n = 1).
(DOCX)

S3 Fig Determination of LCL-derived neuron outgrowth time prior to drug treatment for high content imaging and neurite outgrowth analysis. Allowing 4 h of neurite outgrowth prior to 72 h (a) paclitaxel or (b) vincristine treatment resulted in consistent and the most dramatic dose-response curves across cell lines for relative total outgrowth.
(DOCX)

S4 Fig Determination of siRNA transfection method for gene knockdown in iPSC-derived neurons. The Accell siRNA transfection method showed improved knockdown efficiency over the Dharmafect1 method. iCell Neurons were allowed to grow for 11 days prior to adding either Dharmafect1 (ThermoFisher) transfection media for 5 h (earlier experiments) or Accell (ThermoFisher) transfection media for 24 h (later experiments). The percent of targeted gene remaining was measured 24 h post-transfection in each experiment by qPCR. However, the Dharmafect1 experiments used the RNeasy kit (Qiagen, 10^6^ cells required) and the Accell experiments used the Cells-to-CT kit (Life Technologies, 10^4^ cells required) for RNA isolation. After Dharmafect1 transfection, less than the required number of cells remained for RNA extraction, so the differences between the two methods may be exaggerated. However, since the Accell method was consistently successful, we used it for additional experiments. Each method represents 1 experiment but Accell RNA was in abundance to allow for 3 independent preparations of cDNA for qPCR, as shown.
(DOCX)

S5 Fig Determination of plate coating method for siRNA transfection and high content imaging in iPSC-derived neurons. Dramatic cell loss was observed when using only a single coating of laminin compared to the double-coating method of poly-D-lysine (PDL) plus laminin. Top graphs represent the number of cells available for neurite outgrowth analysis by high content imaging after (a) untransfected-paclitaxel treatment, (b) paclitaxel treatment after Accell transfection of non-targeting control, and (c) vincristine treatment after Accell transfection of non-targeting control. Representative images of 1 well of cells imaged after Accell transfection with the (d) single-coating, laminin only or (e) double-coating plating method using PDL plates and adding cells with laminin.
(DOCX)

S6 Fig Survival and apoptotic response of the LCLs from Coriell that were used to generate iPSCs upon treatment with paclitaxel, vincristine or cisplatin. 1x10^4^ cells/well were (a) exposed to drug for 72 h and assayed for viability using Alamar Blue or (b) exposed to drug for 24 h and assayed for caspase 3/7 activity.
(DOCX)

S7 Fig Intracellular flow cytometry assay for the iPSC-derived neuronal cells to determine purity. The isotype controls from each iPSC-derived neuronal line are on the left. Cortical neurons are defined as Tuj1(βIII-Tubulin)+/Nestin- (gated cells on the right) for each line.
(DOCX)

S1 Movie Time-lapse images (40x) of iCell Neurons treated with 10 μM paclitaxel, 10 μM vincristine, 10 μM cisplatin, 0.17% DMSO vehicle, or 10 μM hydroxyurea taken every 10 min for 28 h. (MOV)

S1 Table Cells available for imaging analysis per well for each experiment. (DOCX)

S2 Table Cell-level neurite outgrowth data of the four chemotherapeutic drug comparison experiments in iCell Neurons. (GZ)

S3 Table Cell-level neurite outgrowth data of the *TUBB2A* siRNA experiments. (GZ)

S4 Table Cell-level neurite outgrowth data of the paclitaxel experiments in four LCL-iPSC-derived neurons. (GZ)

S5 Table Cell-level neurite outgrowth data of the vincristine experiments in four LCL-iPSC-derived neurons. (GZ)

S6 Table Cell-level neurite outgrowth data of the cisplatin experiments in four LCL-iPSC-derived neurons. (GZ)

S7 Table LCL paclitaxel-induced cytotoxicity and caspase-3/7 activity data used to select LCLs for reprogramming into iPSCs. (XLSX)

S8 Table ANOVA results comparing the AUCs of relative neurite outgrowth phenotypes among all 4 drugs or the 3 neurotoxic drugs (Paclitaxel, Vincristine, Cisplatin) after 72 h treatment of iCell Neurons. (DOCX)
