# Supplementary material for: Modeling Chemotherapeutic Neurotoxicity with Human Induced Pluripotent Stem Cell-Derived Neuronal Cells
Source: PLoS One. 2015 Feb 17;10(2):e0118020. doi: 10.1371/journal.pone.0118020 (PMC4331516; doi:10.1371/journal.pone.0118020)
Supplement: S1 Fig — Representative images of iCell Neurons plated at densities of (a) 4 x 10^4^ cells/well and (b) 1.33 x 10^4^ cells/well stained with Calcein AM (green) and analyzed by the MetaXpress software Neurite Outgrowth Application Module (red). Note that at the higher density, clusters of neurons produced regions not accessible for MetaXpress to call cells and measure neurite outgrowths (cells were either missed or given a value of zero for process length measurements). (DOCX) [file pone.0118020.s001.docx]

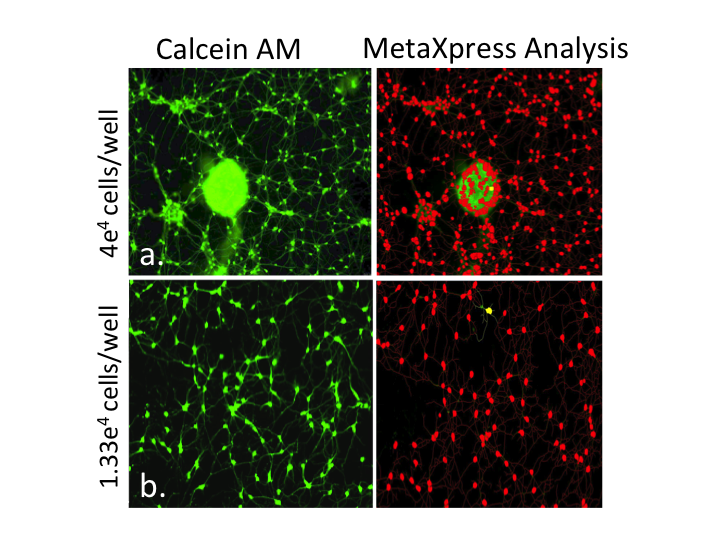


**Fig. S1: Determination of cell density for high content imaging and neurite outgrowth analysis of iPSC-derived neurons.** Representative images of iCell Neurons plated at densities of (a) 4 x 10^4^ cells/well and (b) 1.33 x 10^4^ cells/well stained with Calcein AM (green) and analyzed by the MetaXpress software Neurite Outgrowth Application Module (red). Note that at the higher density, clusters of neurons produced regions not accessible for MetaXpress to call cells and measure neurite outgrowths (cells were either missed or given a value of zero for process length measurements).
