# Supplementary material for: Modeling Chemotherapeutic Neurotoxicity with Human Induced Pluripotent Stem Cell-Derived Neuronal Cells
Source: PLoS One. 2015 Feb 17;10(2):e0118020. doi: 10.1371/journal.pone.0118020 (PMC4331516; doi:10.1371/journal.pone.0118020)
Supplement: S2 Fig — Allowing 4 h of neurite outgrowth prior to 72 h paclitaxel treatment resulted in consistent dose-response curves for relative total outgrowth (n = 3). Allowing 3–11 d of neurite outgrowth prior to either 48 or 72 h PTX treatment did not decrease total outgrowth upon increasing doses (n = 1). Allowing 1 d of neurite outgrowth prior to 48 h PTX treatment may result in consistent dose-response curves for relative total outgrowth, but was only tested once (n = 1). (DOCX) [file pone.0118020.s002.docx]

**
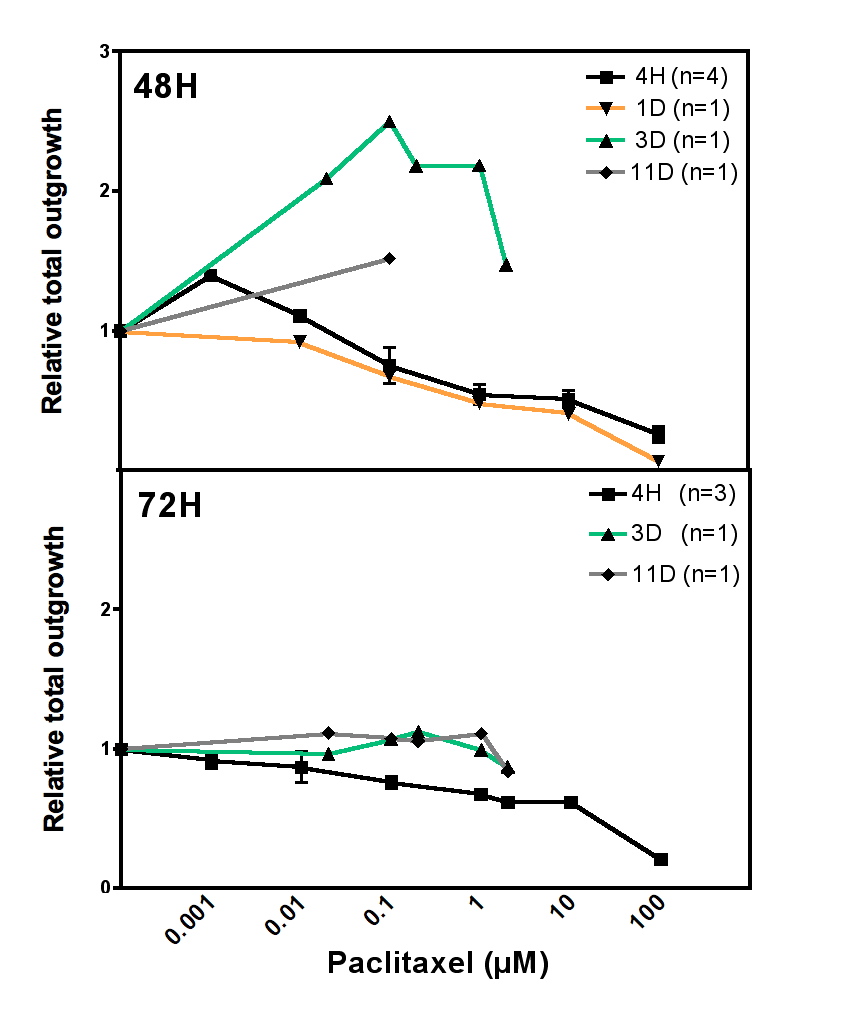
**

**Fig. S2: Determination of iCell Neuron outgrowth time prior to drug treatment for high content imaging and neurite outgrowth analysis.** Allowing 4 h of neurite outgrowth prior to 72 h paclitaxel treatment resulted in consistent dose-response curves for relative total outgrowth (n=3). Allowing 3-11 d of neurite outgrowth prior to either 48 or 72 h PTX treatment did not decrease total outgrowth upon increasing doses (n=1). Allowing 1 d of neurite outgrowth prior to 48 h PTX treatment may result in consistent dose-response curves for relative total outgrowth, but was only tested once (n=1).
