# Supplementary material for: Modeling Chemotherapeutic Neurotoxicity with Human Induced Pluripotent Stem Cell-Derived Neuronal Cells
Source: PLoS One. 2015 Feb 17;10(2):e0118020. doi: 10.1371/journal.pone.0118020 (PMC4331516; doi:10.1371/journal.pone.0118020)
Supplement: S3 Fig — Allowing 4 h of neurite outgrowth prior to 72 h (a) paclitaxel or (b) vincristine treatment resulted in consistent and the most dramatic dose-response curves across cell lines for relative total outgrowth. (DOCX) [file pone.0118020.s003.docx]

**
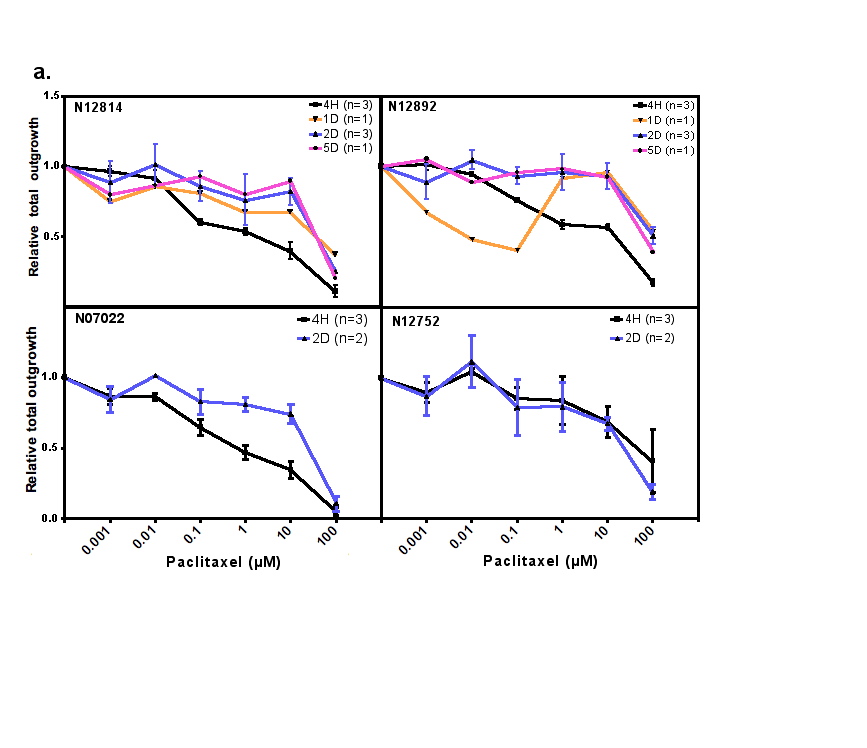

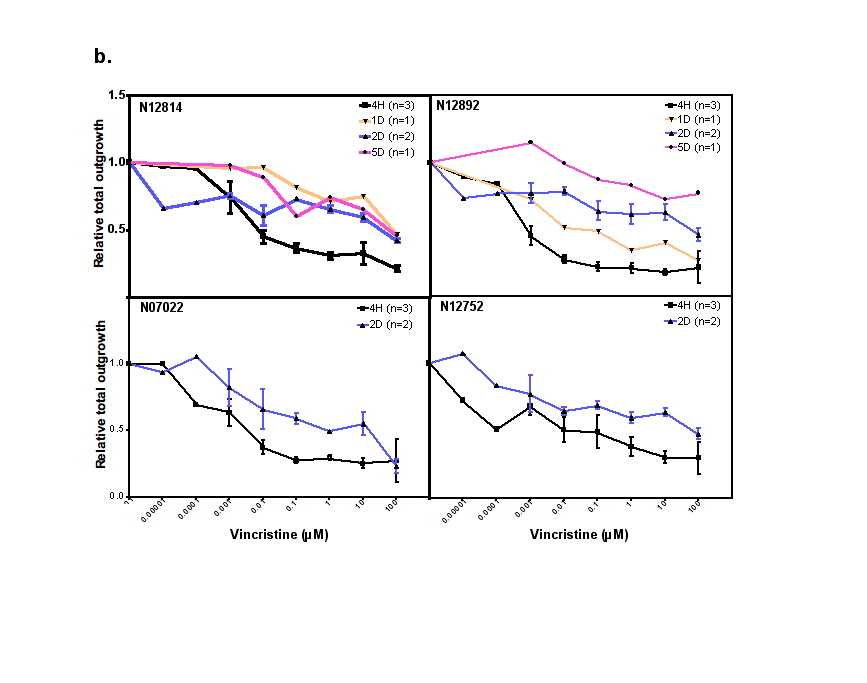
**

**Fig. S3: Determination of LCL-derived neuron outgrowth time prior to drug treatment for high content imaging and neurite outgrowth analysis.** Allowing 4 h of neurite outgrowth prior to 72 h (a) paclitaxel or (b) vincristine treatment resulted in consistent and the most dramatic dose-response curves across cell lines for relative total outgrowth.
