# Supplementary material for: Modeling Chemotherapeutic Neurotoxicity with Human Induced Pluripotent Stem Cell-Derived Neuronal Cells
Source: PLoS One. 2015 Feb 17;10(2):e0118020. doi: 10.1371/journal.pone.0118020 (PMC4331516; doi:10.1371/journal.pone.0118020)
Supplement: S4 Fig — The Accell siRNA transfection method showed improved knockdown efficiency over the Dharmafect1 method. iCell Neurons were allowed to grow for 11 days prior to adding either Dharmafect1 (ThermoFisher) transfection media for 5 h (earlier experiments) or Accell (ThermoFisher) transfection media for 24 h (later experiments). The percent of targeted gene remaining was measured 24 h post-transfection in each experiment by qPCR. However, the Dharmafect1 experiments used the RNeasy kit (Qiagen, 106 cells required) and the Accell experiments used the Cells-to-CT kit (Life Technologies, 104 cells required) for RNA isolation. After Dharmafect1 transfection, less than the required number of cells remained for RNA extraction, so the differences between the two methods may be exaggerated. However, since the Accell method was consistently successful, we used it for additional experiments. Each method represents 1 experiment but Accell RNA was in abundance to allow for 3 independent preparations of cDNA for qPCR, as shown. (DOCX) [file pone.0118020.s004.docx]

**
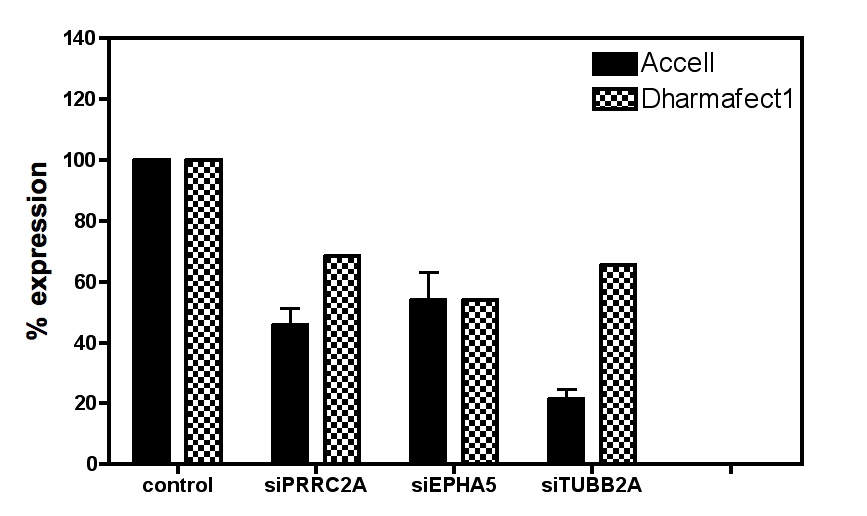
**

**Fig. S4: Determination of siRNA transfection method for gene knockdown in iPSC-derived neurons**. The Accell siRNA transfection method showed improved knockdown efficiency over the Dharmafect1 method. iCell Neurons were allowed to grow for 11 days prior to adding either Dharmafect1 (ThermoFisher) transfection media for 5 h (earlier experiments) or Accell (ThermoFisher) transfection media for 24 h (later experiments). The percent of targeted gene remaining was measured 24 h post-transfection in each experiment by qPCR. However, the Dharmafect1 experiments used the RNeasy kit (Qiagen, 10^6^ cells required) and the Accell experiments used the Cells-to-CT kit (Life Technologies, 10^4^ cells required) for RNA isolation. After Dharmafect1 transfection, less than the required number of cells remained for RNA extraction, so the differences between the two methods may be exaggerated. However, since the Accell method was consistently successful, we used it for additional experiments. Each method represents 1 experiment but Accell RNA was in abundance to allow for 3 independent preparations of cDNA for qPCR, as shown.
