# Supplementary material for: Modeling Chemotherapeutic Neurotoxicity with Human Induced Pluripotent Stem Cell-Derived Neuronal Cells
Source: PLoS One. 2015 Feb 17;10(2):e0118020. doi: 10.1371/journal.pone.0118020 (PMC4331516; doi:10.1371/journal.pone.0118020)
Supplement: S5 Fig — Dramatic cell loss was observed when using only a single coating of laminin compared to the double-coating method of poly-D-lysine (PDL) plus laminin. Top graphs represent the number of cells available for neurite outgrowth analysis by high content imaging after (a) untransfected-paclitaxel treatment, (b) paclitaxel treatment after Accell transfection of non-targeting control, and (c) vincristine treatment after Accell transfection of non-targeting control. Representative images of 1 well of cells imaged after Accell transfection with the (d) single-coating, laminin only or (e) double-coating plating method using PDL plates and adding cells with laminin. (DOCX) [file pone.0118020.s005.docx]

**


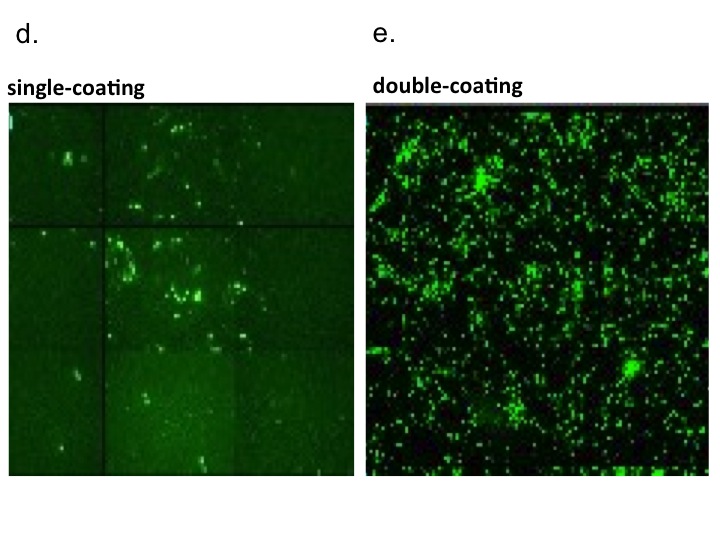
**

**Fig. S5: Determination of plate coating method for siRNA transfection and high content imaging in iPSC-derived neurons.** Dramatic cell loss was observed when using only a single coating of laminin compared to the double-coating method of poly-D-lysine (PDL) + laminin. Top graphs represent the number of cells available for neurite outgrowth analysis by high content imaging after (a) untransfected-paclitaxel treatment, (b) paclitaxel treatment after Accell transfection of non-targeting control, and (c) vincristine treatment after Accell transfection of non-targeting control. Representative images of 1 well of cells imaged after Accell transfection with the (d) single-coating, laminin only or (e) double-coating plating method using PDL plates and adding cells with laminin.
