# Supplementary material for: Modeling Chemotherapeutic Neurotoxicity with Human Induced Pluripotent Stem Cell-Derived Neuronal Cells
Source: PLoS One. 2015 Feb 17;10(2):e0118020. doi: 10.1371/journal.pone.0118020 (PMC4331516; doi:10.1371/journal.pone.0118020)
Supplement: S6 Fig — 1x10^4^ cells/well were (a) exposed to drug for 72 h and assayed for viability using Alamar Blue or (b) exposed to drug for 24 h and assayed for caspase 3/7 activity. (DOCX) [file pone.0118020.s006.docx]

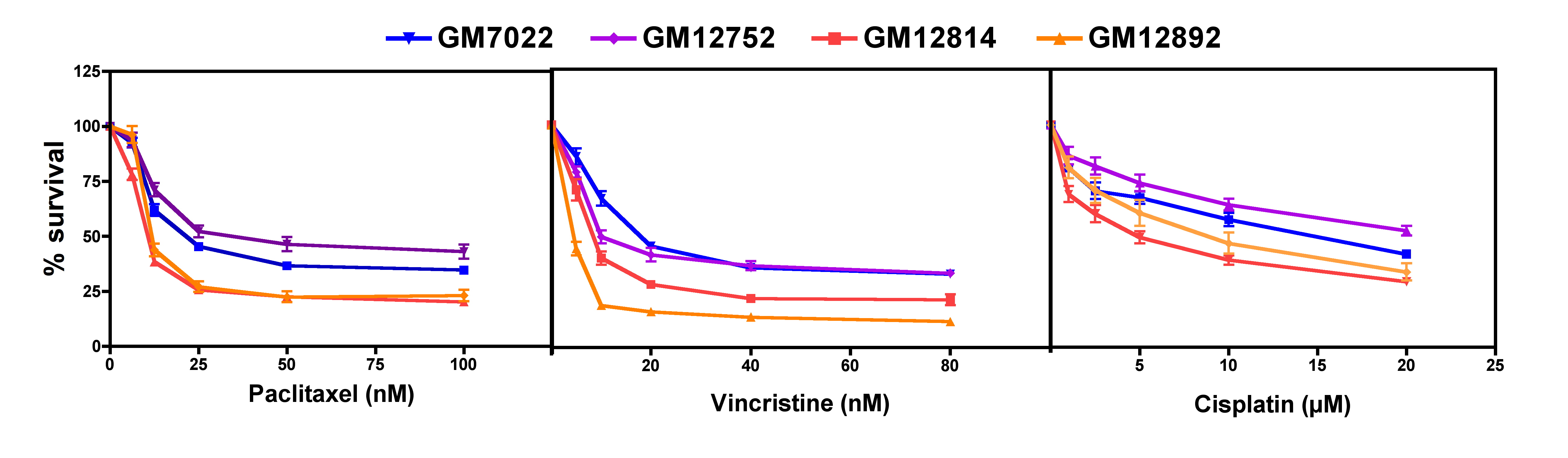


a

**
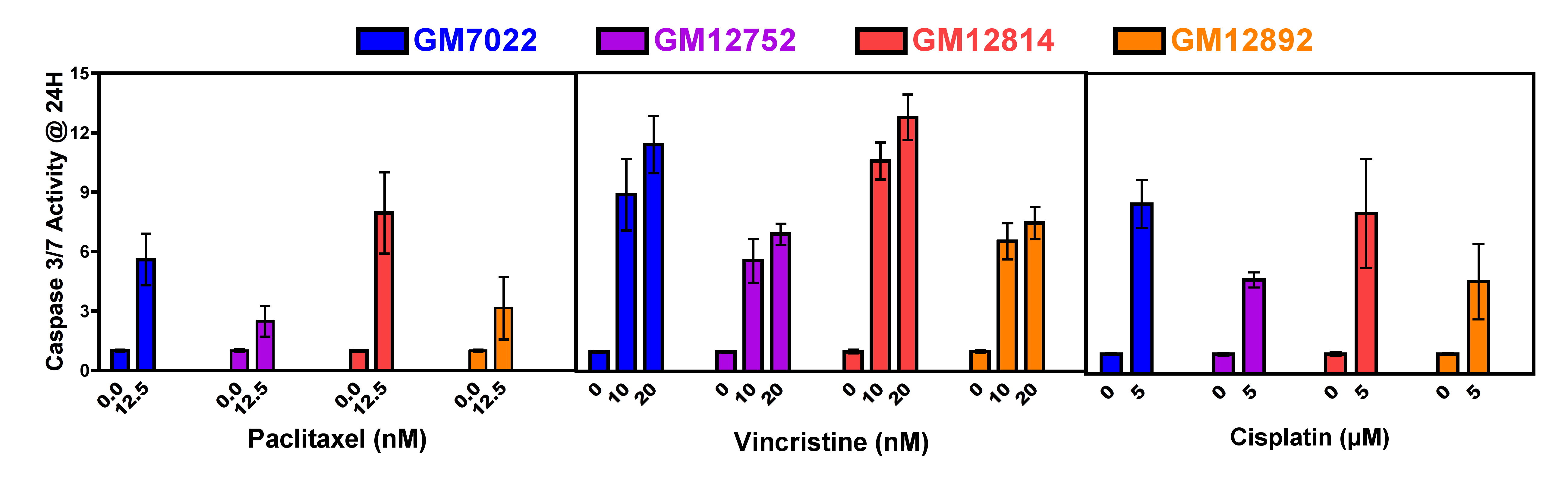
**

b

**Fig. S6: Survival and apoptotic response of the LCLs from Coriell that were used to generate iPSCs upon treatment with paclitaxel, vincristine or cisplatin.** 1x10^4^ cells/well were (a) exposed to drug for 72 h and assayed for viability using Alamar Blue or (b) exposed to drug for 24 h and assayed for caspase 3/7 activity.
