# Supplementary material for: Modeling Chemotherapeutic Neurotoxicity with Human Induced Pluripotent Stem Cell-Derived Neuronal Cells
Source: PLoS One. 2015 Feb 17;10(2):e0118020. doi: 10.1371/journal.pone.0118020 (PMC4331516; doi:10.1371/journal.pone.0118020)
Supplement: S7 Fig — The isotype controls from each iPSC-derived neuronal line are on the left. Cortical neurons are defined as Tuj1(βIII-Tubulin)+/Nestin- (gated cells on the right) for each line. (DOCX) [file pone.0118020.s007.docx]

N07022


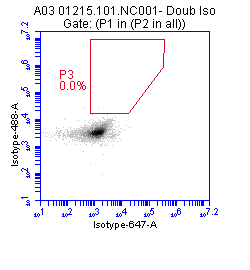

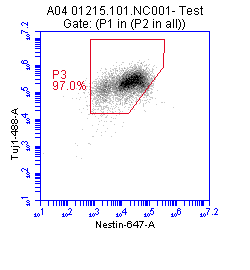


N12752


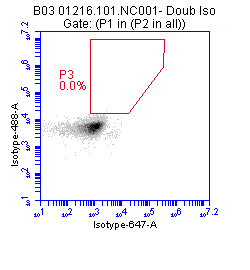

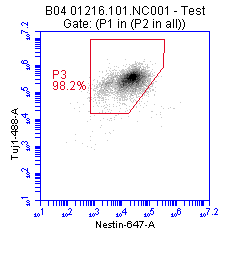


N12814


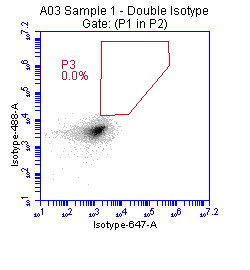

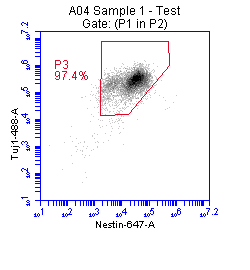


N12892


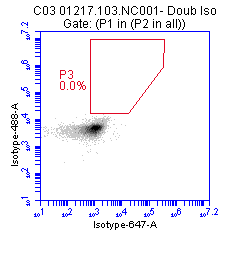

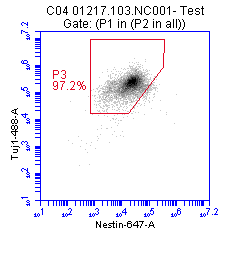


**Figure S7. Intracellular flow cytometry assay for the iPSC-derived neuronal cells to determine purity.** The isotype controls from each iPSC-derived neuronal line are on the left. Cortical neurons are defined as Tuj1(βIII-Tubulin)+/Nestin- (gated cells on the right) for each line.
