# Supplementary material for: Modeling Chemotherapeutic Neurotoxicity with Human Induced Pluripotent Stem Cell-Derived Neuronal Cells
Source: PLoS One. 2015 Feb 17;10(2):e0118020. doi: 10.1371/journal.pone.0118020 (PMC4331516; doi:10.1371/journal.pone.0118020)
Supplement: S1 Table — (DOCX) [file pone.0118020.s009.docx]

**Table S1: Cells available for imaging analysis per well for each experiment.**

Four drug treatment comparison using iCell Neurons.

| (µM) | Paclitaxel | | | Vincristine | | | Cisplatin | | | Hydroxyurea | | |
| --- | --- | --- | --- | --- | --- | --- | --- | --- | --- | --- | --- | --- |
|  | Expt. 1 Expt.2 Expt. 3 | | | Expt. 1 Expt. 2 Expt. 3 | | | Expt. 1 Expt. 2 Expt. 3 | | | Expt. 1 Expt. 2 Expt. 3 | | |
| 0 | 735 | 747 | 736 | 857 | 726 | 864 | 706 | 1547 | 1276 | 538 | 723 | 1221 |
| 0.001 | - | 615 | 450 | - | 753 | 733 | 852 | 1341 | 1070 | - | 727 | 734 |
| 0.01 | - | 623 | 796 | - | 780 | 569 | 789 | 1334 | 1268 | - | 665 | 880 |
| 0.1 | 758 | 764 | 960 | 791 | 670 | 504 | 736 | 1259 | 1260 | 398 | 724 | 788 |
| 1 | 689 | 788 | 967 | 587 | 709 | 467 | 814 | 1374 | 958 | 508 | 833 | 806 |
| 10 | 710 | 589 | 1076 | 887 | 624 | 541 | 256 | 222 | 735 | 398 | 795 | 881 |
| 100 | 217 | 232 | 418 | 206 | 198 | - | - | 113 | 165 | 594 | 790 | 851 |

Four LCL-derived neuron comparison.

| (µM) | | N07022 | | | N12752 | | | N12814 | | | N12892 | | |
| --- | --- | --- | --- | --- | --- | --- | --- | --- | --- | --- | --- | --- | --- |
|  |  | Expt.1 | Expt.2 | Expt.3 | Expt.1 | Expt.2 | Expt.3 | Expt.1 | Expt.2 | Expt.3 | Expt.1 | Expt.2 | Expt.3 |
| Paclitaxel | 0 | 1229 | 1145 | 1188 | 879 | 897 | 1227 | 1043 | 1488 | 1511 | 1023 | 1533 | 1607 |
|  | 0.001 | 1198 | 1008 | 1194 | 792 | 643 | 1226 | 1044 | 1706 | 1507 | 704 | 901 | 1661 |
|  | 0.01 | 1029 | 1027 | 1143 | 724 | 552 | 1543 | 1106 | 1863 | 1449 | 595 | 980 | 1685 |
|  | 0.1 | 1052 | 1028 | 1117 | 814 | 200 | 1217 | 1109 | 1949 | 1142 | 683 | 859 | 1601 |
|  | 1 | 1109 | 1066 | 1161 | 711 | 610 | 1386 | 1016 | 2056 | 1369 | 475 | 1280 | 1642 |
|  | 10 | 832 | 1013 | 1032 | 442 | 396 | 1244 | 821 | 2158 | 1324 | 250 | 1139 | 1525 |
|  | 100 | 277 | 468 | 71 | 60 | 593 | 349 | 454 | 1304 | 343 | 43 | 815 | 359 |
| Vincristine | 0 | 1076 | 1162 | 816 | 757 | 713 | 716 | 919 | 861 | 891 | 897 | 1442 | 1302 |
|  | 0.001 | 365 | 882 | 887 | 370 | 918 | 929 | 1218 | 878 | 1274 | 834 | 1328 | 1351 |
|  | 0.01 | 789 | 1080 | 919 | 1119 | 819 | 828 | 570 | 524 | 1058 | 785 | 1197 | 1358 |
|  | 0.1 | 248 | 955 | 989 | 1114 | 1042 | 811 | 1314 | 592 | 1131 | 789 | 1452 | 1274 |
|  | 1 | 457 | 1106 | 994 | 542 | 1168 | 890 | 1636 | 584 | 1135 | 837 | 1666 | 1293 |
|  | 10 | 789 | 748 | 806 | 507 | 881 | 835 | 738 | 549 | 1197 | 729 | 1571 | 1151 |
|  | 100 | 154 | 520 | 434 | 150 | 1267 | 393 | 513 | 783 | 727 | 168 | 648 | 766 |
| Cisplatin | 0 | 985 | 1041 | 1290 | 742 | 769 | 1675 | 1636 | 1475 | 1646 | 708 | 1296 | 1554 |
|  | 0.001 | 1061 | - | 1344 | 121 | 1034 | 1537 | 1724 | 1029 | 937 | 177 | 1555 | 1510 |
|  | 0.01 | 848 | 1155 | 1387 | 404 | 868 | 1629 | 1680 | 1150 | 1136 | 292 | 1423 | 1756 |
|  | 0.1 | 1337 | 1239 | 1263 | 937 | 1033 | 1559 | 1692 | 1429 | 1028 | 478 | 899 | 1671 |
|  | 1 | 991 | 889 | 1123 | 715 | 1009 | 1179 | 1679 | 1227 | 981 | 513 | 1842 | 1540 |
|  | 10 | 536 | 510 | 437 | 355 | 805 | 492 | 723 | 460 | 289 | 352 | 774 | 537 |
|  | 100 | 164 | 202 | 215 | 112 | 637 | 241 | 651 | 435 | 318 | 221 | 189 | 413 |

TUBB2A siRNA transfection experiments.

| Paclitaxel (µM) | Non-targeting control | | | siTUBB2A | | |
| --- | --- | --- | --- | --- | --- | --- |
|  | Expt. 1 | Expt. 2 | Expt. 3 | Expt. 1 | Expt. 2 | Expt. 3 |
| 0 | 1642 | 1346 | 678 | 1587 | 650 | 461 |
| 0.1 | 1771 | 1572 | 999 | 1717 | 1134 | 917 |
