# Supplementary material for: Modeling Chemotherapeutic Neurotoxicity with Human Induced Pluripotent Stem Cell-Derived Neuronal Cells
Source: PLoS One. 2015 Feb 17;10(2):e0118020. doi: 10.1371/journal.pone.0118020 (PMC4331516; doi:10.1371/journal.pone.0118020)
Supplement: S8 Table — (DOCX) [file pone.0118020.s016.docx]

**Table S8. ANOVA results comparing the AUCs of relative neurite outgrowth phenotypes among all 4 drugs or the 3 neurotoxic drugs (Paclitaxel, Vincristine, Cisplatin) after 72 h treatment of iCell Neurons**

| Phenotype | Paclitaxel AUC (SE) | Vincristine AUC (SE) | Cisplatin AUC (SE) | Hydroxyurea AUC (SE) | 4 drug ANOVA  P value | 3 neurotoxic drug ANOVA  P value |
| --- | --- | --- | --- | --- | --- | --- |
| total outgrowth | 5.93 (0.01) | 5.43 (0.17) | 5.46 (0.24) | 6.68 (0.06) | 0.003 | 0.119 |
| number of processes | 6.42 (0.05) | 6.53 (0.12) | 5.80 (0.13) | 6.67 (0.04) | 0.017 | 0.043 |
| mean process length | 6.11 (0.02) | 5.37 (0.08) | 5.69 (0.19) | 6.68 (0.04) | 0.0007 | 0.009 |
| median process length | 6.18 (0.005) | 5.39 (0.06) | 5.74 (0.17) | 6.69 (0.03) | 0.001 | 0.005 |
| maximum process length | 5.99 (0.04) | 5.34 (0.12) | 5.54 (0.24) | 6.68 (0.05) | 0.002 | 0.040 |
| number of branches | 5.80 (0.03) | 5.54 (0.32) | 4.91 (0.36) | 6.71 (0.08) | 0.004 | 0.226 |
| straightness | 6.56 (0.01) | 6.46 (0.04) | 6.05 (0.07) | 6.67 (0.03) | 0.007 | 0.013 |
| cell body area | 6.66 (0.08) | 6.68 (0.02) | 6.53 (0.02) | 6.67 (0.05) | 0.045 | 0.030 |
| mean outgrowth intensity | 6.90 (0.01) | 6.59 (0.04) | 5.92 (0.18) | 6.66 (0.04) | 0.005 | 0.009 |

ANOVA = one-way analysis of variance (not assuming equal variances), SE = standard error, AUC = area under the concentration curve calculated from 0.001-100 µM
